# Supplementary material for: Vascular defects of DYRK1A knockouts are ameliorated by modulating calcium signaling in zebrafish
Source: Dis Model Mech. 2019 May 23;12(5):dmm037044. doi: 10.1242/dmm.037044 (PMC6550036; doi:10.1242/dmm.037044)
Supplement: Supplementary information [file dmm-12-037044-s1.pdf]

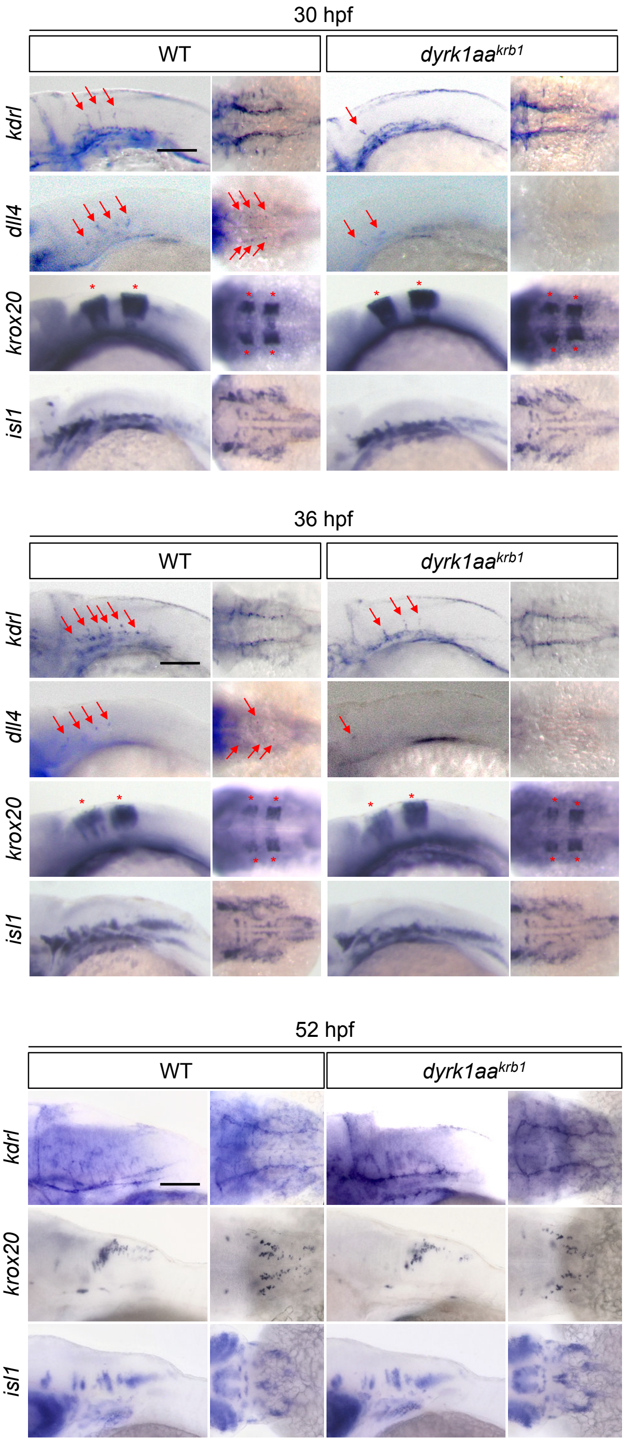

Figure S1. **Expression of vascular markers is reduced in *dyrk1aa*^{krb1} mutant embryos, but the hindbrain development is not affected.** The vascular markers of *kdrl* and *dll4* were reduced at 30 and 36 hpf (red arrows), and *kdrl* expression was decreased in 52 hpf. The markers of *krox20* (red asterisks) and *isl1* were unchanged in *dyrk1aa*^{krb1} mutant embryos. Scale bars: 100 µm

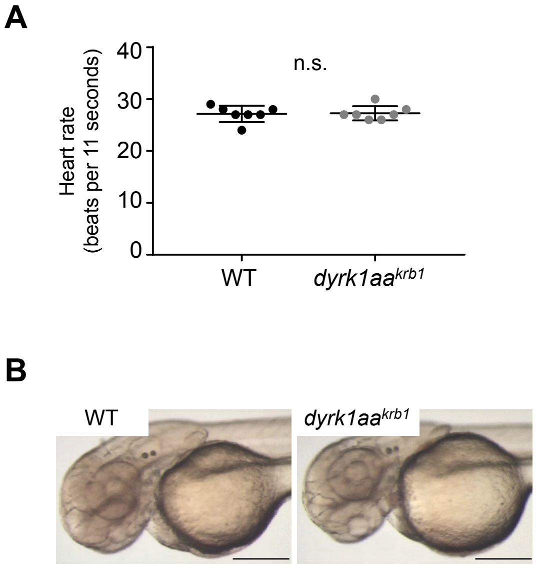

Figure S2. ***dyrk1aa*** **mutation does not affect the heart rates of zebrafish embryos at 52 hpf.** (A) Heart rates were measured in WT and *dyrk1aa*^{krb1} embryos under a light microscope during 11 seconds at RT. *N*=7 each genetic group. (B) The captured images to measure the heart rates of WT and *dyrk1aa*^{krb1} embryos (refer to the Movies 1 and 2 for actual movies). *p*-values by Mann-Whitney U test: n.s., not significant. Data are mean ±s.e.m. Scale bars: 250 µm

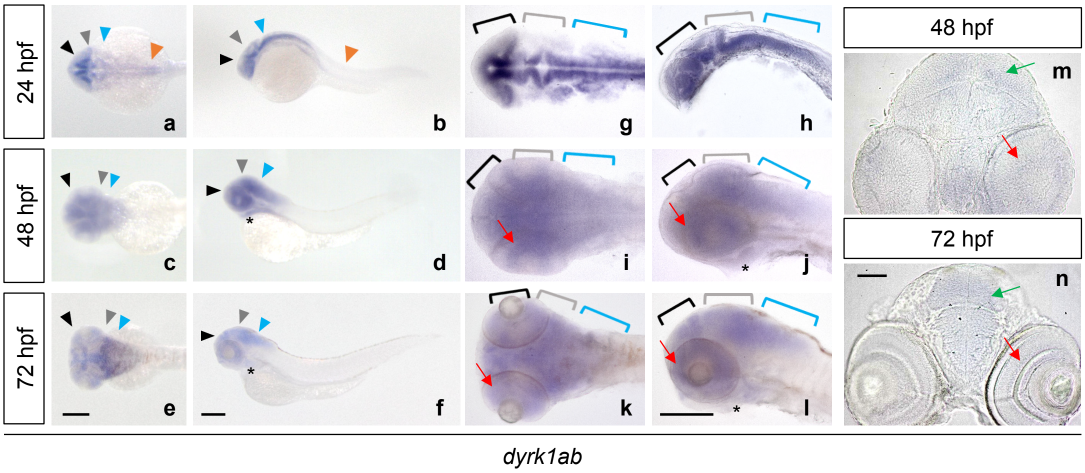

Figure S3. ***dyrk1ab* is expressed in the developing brain region.** By WISH (whole mount in situ hybridization), *dyrk1ab* was expressed in the forebrain (black arrowheads, a-f; black brackets, g-l), the midbrain (gray arrowheads, a-f; gray brackets, g-l), the hindbrain (blue arrowheads, a-f; blue brackets, g-l) at 24, 48 and 72 hpf and the spinal cord (orange arrowheads, a and b) at 24 hpf. It was also detected in the heart (asterisks, d, f, j and l) and in the retina (red arrows, i-l) at 48 and 72 hpf. (m and n) Sectioned images of WISH embryos showed the expression of *dyrk1ab* in the tectum (green arrows) and the retina (red arrows) at 48 hpf and 72 hpf. Scale bars: 200 µm in (a-l) and 50 µm in (m and n)

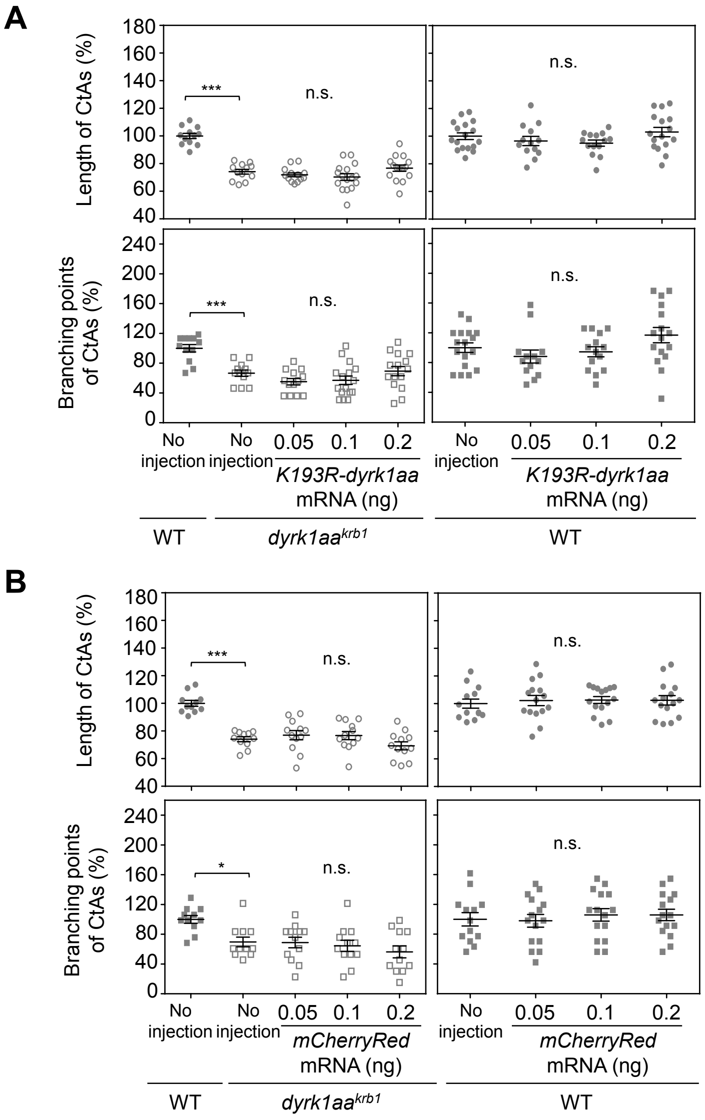

Figure S4. The CtA development of WT and *dyrk1aa*^{krb1} embryos is not affected by *K193R-dyrk1aa* and *mCherryRed* mRNA injection. No differences were observed in the mean percentages of length and branching points of CtAs in WT and *dyrk1aa*^{krb} embryos (52 hpf) by (A) *K193R-dyrk1aa* and (B) *mCherryRed* mRNA injection as control. $N \geq 11$ each group. p-values by one-way ANOVA: *, $p<0.05$ and ***, $p<0.005$; n.s., not significant. Data are mean±s.e.m.

Disease Models & Mechanisms • Supplementary information

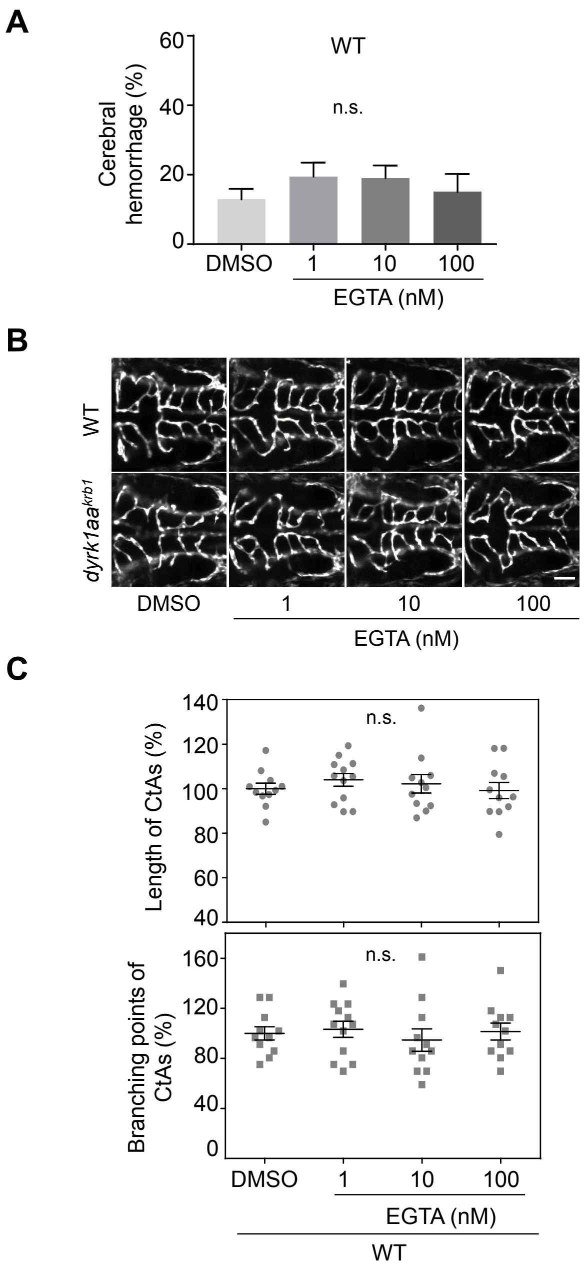

Figure S5. **The vascular phenotype by EGTA treatment is not affected in WT embryos.** No differences were observed in the mean percentages of cerebral hemorrhage in WT embryos (A) and the mean percentages of length and branching points of CtAs (C) by 1, 10 and 100 nM EGTA treatment. (B) The compiled images of CtAs by confocal microscopy show that the development of the CtAs with *dyrk1aa*^{krb1} embryos are rescued by 1 nM and 10 nM of EGTA treatment in the *Tg*(*kdrl:EGFP*) background (see Fig. 6D). WT embryos are not affected by EGTA treatment. *p*-values by one-way ANOVA: n.s., not significant. Data are mean±s.e.m. Scale bar: 50 µm.

Disease Models & Mechanisms • Supplementary information

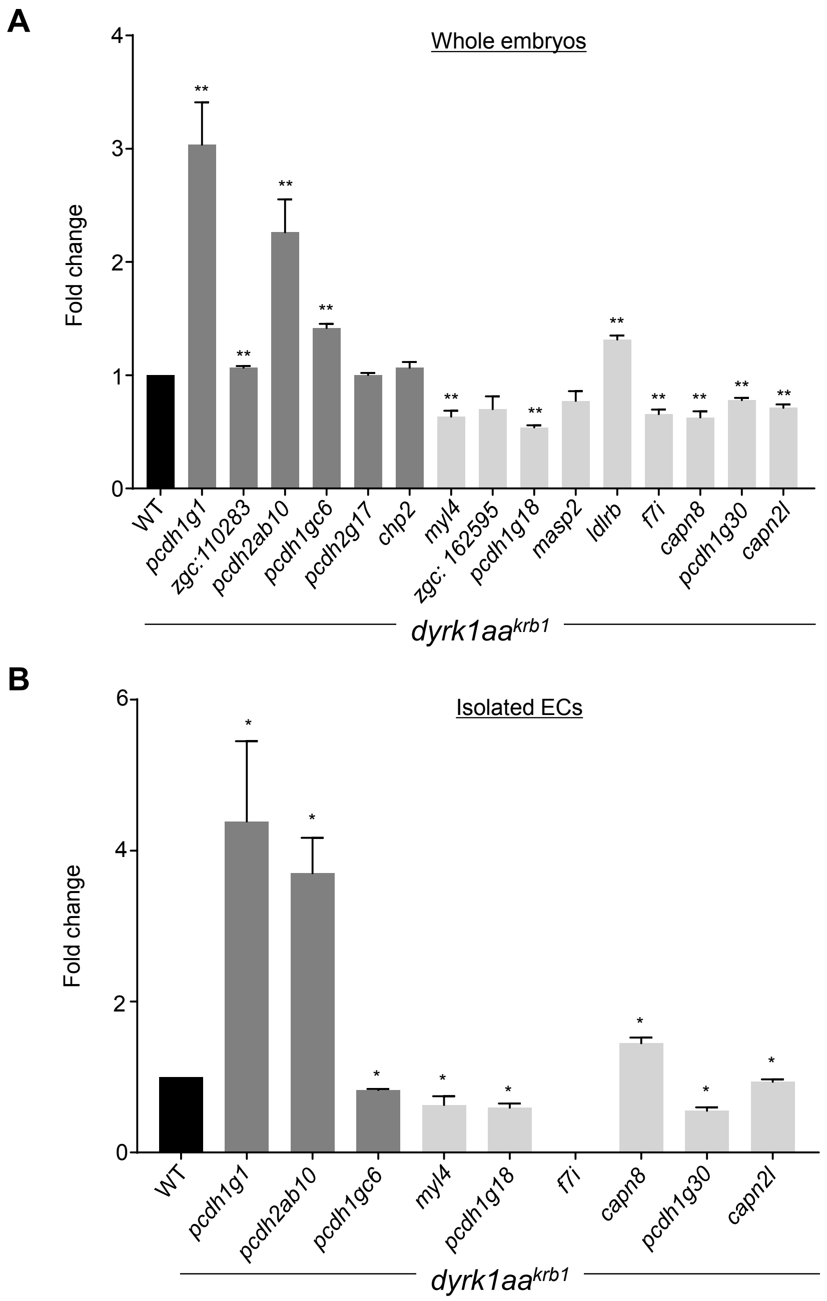

Figure S6. **The validation of calcium ion binding genes by real-time qRT-PCR.** (A) 15 "calcium ion binding" GO category genes were validated in whole embryos of *dyrk1aa*^{krb1} compared to WT as shown in the graph. Pooled 20 embryos of each of WT and *dyrk1aa*^{krb1} (the number of biological repeats=2) were analyzed by real-time qRT-PCR. The expression alterations of 10 genes (*pcdh1g1, zgc: 110283, pcdh2ab10, pcdh1gc6, myl4, pcdh1g18, f7i, capn8, pcdh1g30* and *capn2*l) in "calcium ion binding" category were confirmed with two genes (*zgc: 162595* and *masp2*) altered but not statistically significant, and three genes (*pcdh2g17, chp2* and *ldlrb*) not recapitulating the transcriptomic analysis (see Fig. 7). The dark gray bars indicate the differentially up-regulated genes and the light gray bars indicate the differentially down-regulated genes by whole transcriptomic analysis of 48 hpf. (B) The graph showing the verification of expression of nine genes using RNAs from endothelial cells by qRT-PCR. The GFP-positive endothelial cells of each WT and *dyrk1aa*^{krb1} were isolated by FACS (the number of biological repeats=2) and analyzed by realtime qRT-PCR. Seven genes (*pcdh1g1, pcdh2ab10, myl4, pcdh1g18, f7i, pcdh1g30* and *capn2l*), except the *capn8* and *pcdh1gc6* genes, exhibited the similar expression patterns in endothelial cells as in the whole embryos. The fold changes of DEGs in *dyrk1aa*^{krb1} were calculated based on the WT level (black bars). qRT-PCR and isolating endothelial cells were performed by the same Materials and Methods of "RNA preparation and RT-PCR analysis", and "FACS analysis of endothelial cells", respectively, in the main text (See Materials and Methods in Main text for more information). *p*- values by Mann-Whitney U test: *, $p<0.05$; **, $p<0.01$. Data are mean±s.e.m.

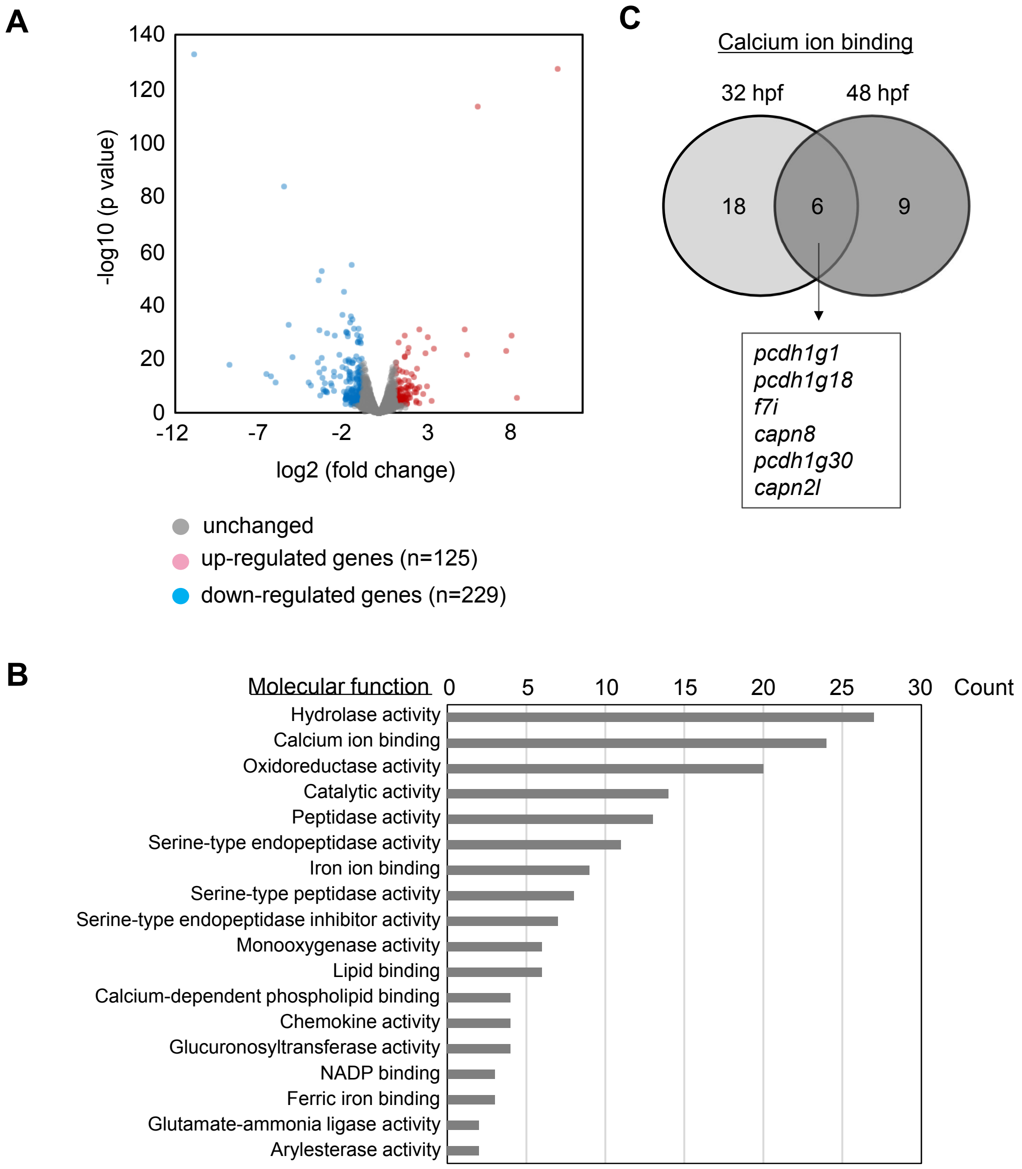

Figure S7. **The whole transcriptomic analysis of *dyrk1aa*^{krb1} compared with WT at 32 hpf.** (A) The volcano plot showing the whole transcriptomes of *dyrk1aa*^{krb1} and the differentially up- (red dots, n=125) or down- (blue dots, n=229) expressed genes (DEGs, more than 2 fold, p<0.05) in *dyrk1aa*^{krb1} embryos compared to WT. The transcriptomic analysis was performed by pooling 20 embryos of each of WT and *dyrk1aa*^{krb1} at 32 hpf (the number of biological repeats=2). (B) A bar graph showing the list of groups of DEGs in the classification of the molecular function. The DEGs belonging to "Hydrolase activity" and "calcium ion binding" groups were most significant at 32 hpf. The results of RNA-seq data were deposited in NCBI (GEO: GSE123026).This experiment was performed by the same Materials and Methods of "Isolation, Library preparation and sequencing for RNA seq" except using RNAs from 32 hpf embryos (See Materials and Methods in Main text for more information). (C) The diagram showing DEGs of the "calcium ion binding" which was commonly altered at 32 hpf and 48 hpf.

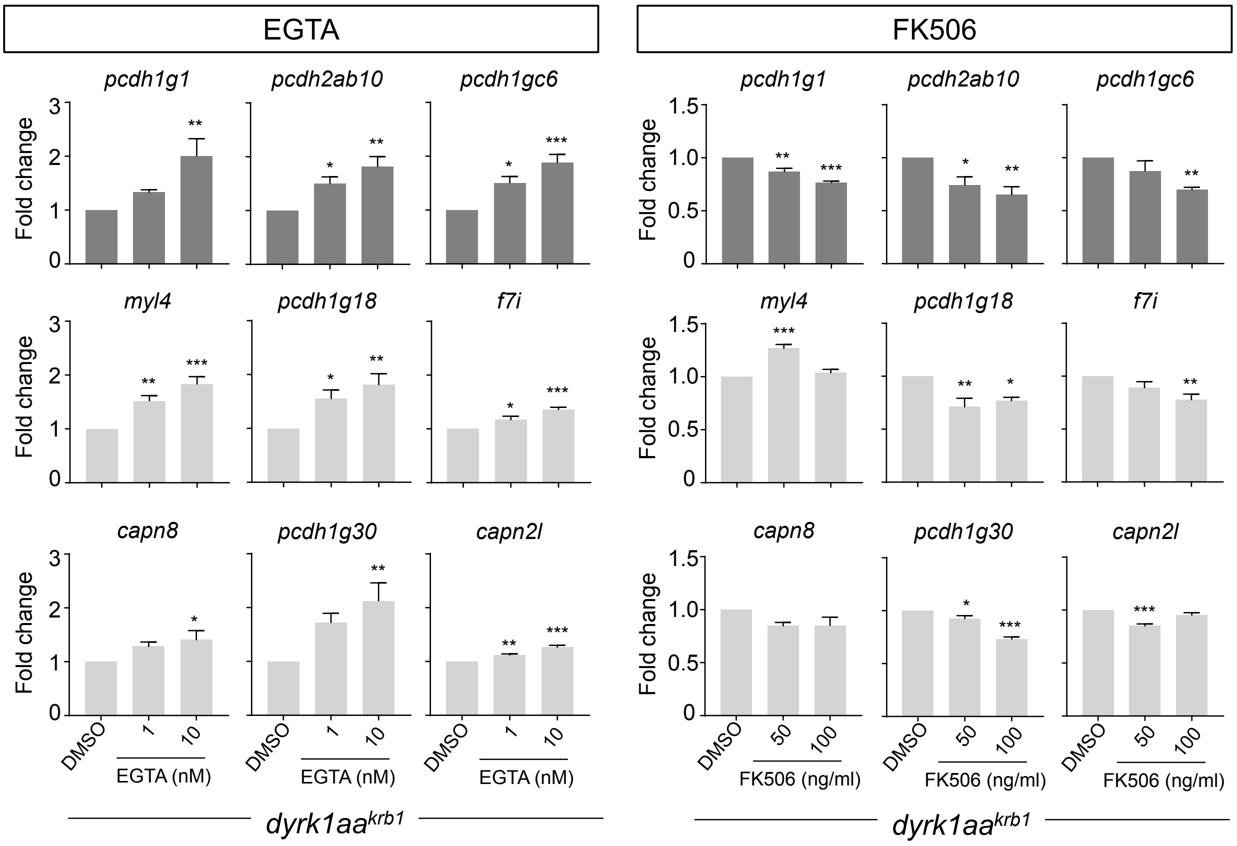

Figure S8. **Expression changes of the calcium ion binding genes upon EGTA and FK506 treatment.** The alterations of nine genes (*pcdh1g1, pcdh2ab10, pcdh1gc6, myl4, pcdh1g18, f7i, capn8, pcdh1g30* and *capn2l)* in the "calcium ion binding" category by drug treatments was validated by qRT-PCR using the whole embryo RNA of each of WT and *dyrk1aa^{krb1}*. Although some expression changes of 9 genes were observed, they did not consistently reflect the phenotypic rescues upon drug treatment. The dark gray bars indicate the differentially up-regulated genes and the light gray bars indicate the differentially down-regulated genes by whole transcriptomic analysis of 48 hpf. The fold change was calculated based on each DMSO control. The drug treatment was performed under the same conditions and concentrations that were confirmed to show the rescue effects of vascular phenotypes (CtA development and hemorrhagic phenotype) in *dyrk1aa^{krb1}* (see Fig. 6 for EGTA treatment, Fig. 8 for FK506 treatment and "Chemical treatment and small molecule library screening" in Materials and Methods). qRT-PCR was done by "RNA preparation and RT-PCR analysis" in Materials and Methods. *p*-values by one-way ANOVA: *, $p<0.05$; **, $p<0.01$; ***, $p<0.005$. Data are mean±s.e.m.

Disease Models & Mechanisms • Supplementary information

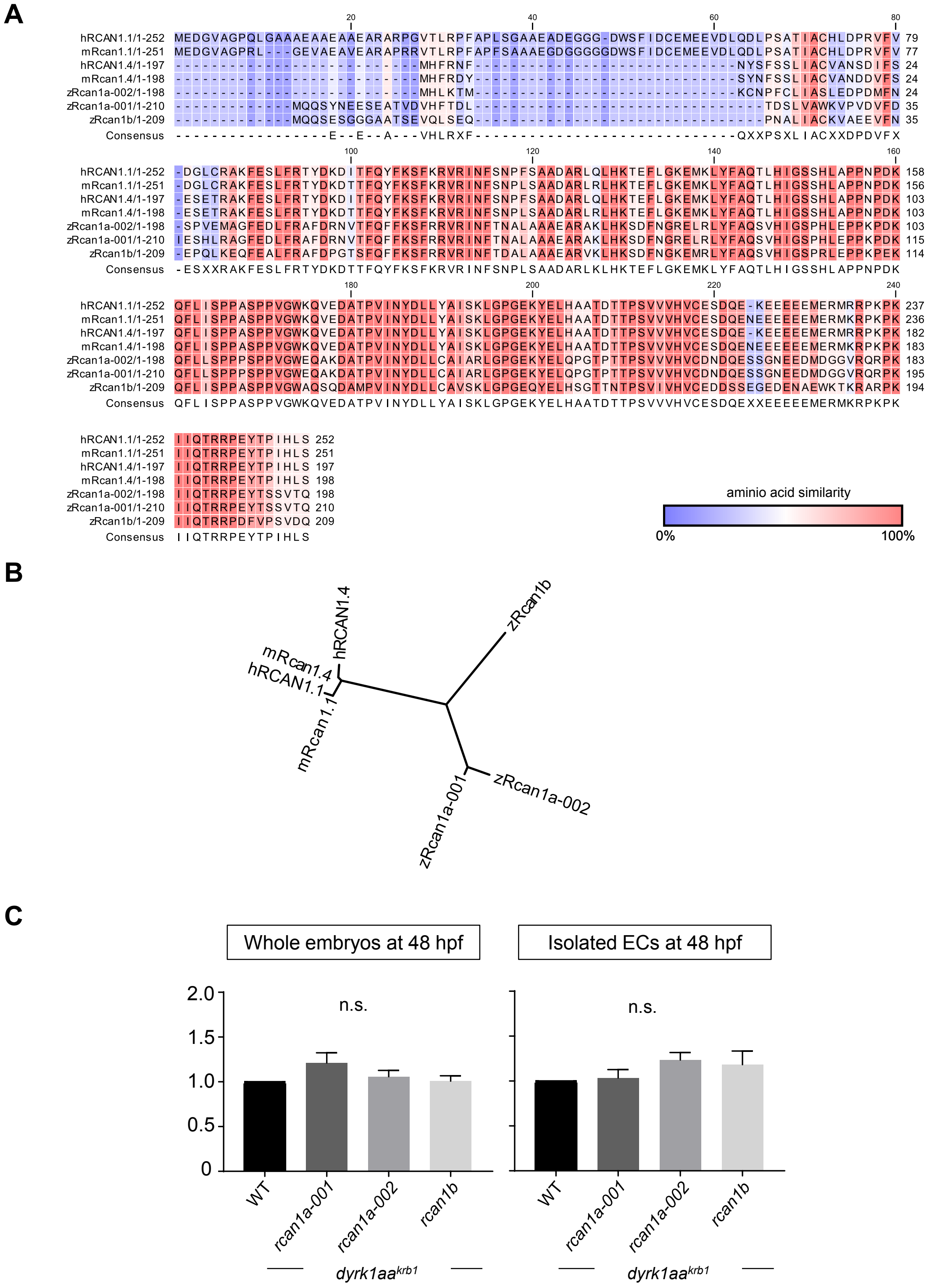

Figure S9. **Identification of zebrafish *rcan1a-001, rcan1a-002*, and *rcan1b* and comparative analyses of their expression in whole embryos and isolated endothelial cells (ECs).** (A) Amino acid sequence alignments of zebrafish Rcan1a-001 (NP_001003616), Rcan1a-002 (XP_005167884), and Rcan1b (XP_021326477), compared with human RCAN1.1 (NP_004405), murine RCAN1.1 (NP_001075018), human RCAN1.4 (NP_981963) and murine RCAN1.4 (AAF72701). (B) A phylogenic tree of zebrafish Rcan1a-001, Rcan1a-002, and Rcan1b, together with human and murine RCAN1.1 and RCAN1.4. (C) Expression of zebrafish *rcan1a-001, rcan1a-002*, and *rcan1b* was not changed in *dyrk1aa*^{krb1} mutant RNAs of whole embryos and isolated ECs at 48 hpf by qRT-PCR analysis. The fold change was calculated based on levels of WT control. qRT-PCR was done by "RNA preparation and RT-PCR analysis" in Materials and Methods. *p*- values by Mann-Whitney U test: n.s., not significant. Data are mean±s.e.m.

Table S1. The table shows the classes of 1280 LOPAC library and results of *in vivo* chemical screening using hemorrhagic phenotype of zebrafish embryos. The Screened Ratio was calculated by Total Screened / Total Agents x 100 (%).

Class	Total Agents	Increased	Reduced	Total Screened	Screened Ratio (%)
Antioxidants	1	—	1	1	100.00
Adenosine	41	7	—	7	17.07
Adrenoceptor	91	8	4	12	13.19
Angionenesis	5	1	—	1	20.00
Antibiotic	29	1	2	3	10.34
Anticonvulsant	10	2	—	2	20.00
Apoptosis	29	2	2	4	13.79
Bioactive Small Molecules	1	—	1	1	100.00
Biochemistry	42	4	2	6	14.29
Ca^{2+} Channel	21	1	—	1	4.76
Cell cycle	18	3	1	4	22.22
Cell signaling	4	1	—	1	25.00
Cell stress	19	3	1	4	21.05
Cholinergic	72	7	3	10	13.89
Cl^- Channel	3	1	—	1	33.33
Cyclic Nucleotides	30	—	2	2	6.67
DNA	23	3	1	4	17.39
Dopamine	92	9	3	12	13.04
GABA	36	4	—	4	11.11
Gene regulation	13	1	—	1	7.69
Glutamate	79	6	4	10	12.66
G-Protein	3	1	—	1	33.33
Histamine	35	2	4	6	17.14
Hormone	29	3	—	3	10.34
Immune signaling	20	5	3	8	40.00
Intracellular calcium	6	—	1	1	16.67
Ion channel	27	2	—	2	7.41
K^+ channel	25	3	—	3	12.00
Kinase/phostpatase	11	2	—	2	18.18
Leukotriene	6	1	—	1	16.67
Lipid	21	4	—	4	19.05
Melatonin	7	—	1	1	14.29
Multi-Drug Resistance	11	1	—	1	9.09
Na^+ Channel	18	3	1	4	22.22
Neuroscience	3	1	—	1	33.33
Neurotrasmission	64	3	3	6	9.38
Nitric oxide	28	5	—	5	17.86
Opioid	20	1	1	2	10.00
P2 Receptor	11	1	—	1	9.09
Phospholyation	88	11	—	11	12.50
Prostaglandin	24	4	—	4	16.67
Serotonin	81	8	2	10	12.35
Transcription	12	2	—	2	16.67
Tyrosine Kinase	4	1	—	1	25.00
Vanilloid	4	—	—	—	0.00
Autophagy	1	—	—	—	0.00
Benzodiazepine	3	—	—	—	0.00
Sigma receptor	1	—	—	—	0.00
Somatostatin	1	—	—	—	0.00
Sphingolipid	4	—	—	—	0.00
Tachykinin	4	—	—	—	0.00
Thromboxane	1	—	—	—	0.00
Purinergics	1	—	—	—	0.00
Purinoceptor	2	—	—	—	0.00
Phosphodiesterase	4	—	—	—	0.00
Nootropic	3	—	—	—	0.00
Muscarinic	1	—	—	—	0.00
Excitatory Amino Acids	1	—	—	—	0.00
Adrenergics	3	—	—	—	0.00
Anticoagulants	1	—	—	—	0.00
Cell Biology	3	—	—	—	0.00
Cholecystokinin	2	—	—	—	0.00
Cytokine	2	—	—	—	0.00
Cytoskeleton	13	—	—	—	0.00
Glycine	2	—	—	—	0.00
Imidazoline	10	—	—	—	0.00
Total	1280	128	43	171	13.36

Table S2. The table shows the small molecule candidates of 128 that were identified to increase the brain hemorrhagic phenotype. The intensity of red color represents the degree of increase: the pale red color indicates mild increase while the dark red color does a strong increase.

Class	Name	Symptom	Degree of Increase
Adenosine	Chloro-IB-MECA	toxic	
	Aminophylline ethylenediamine		
	1,3-Dipropyl-8-p-sulfophenylxanthine		
	1-Allyl-3,7-dimethyl-8-p-sulfophenylxanthine	toxic	
	CGS-15943	toxic/ heart edema/ heart beat defect	
	3,7-Dimethyl-1-propargylxanthine		
	Dipyridamole		
Adrenoceptor	Formoterol		
	(-)-Isoproterenol hydrochloride	toxic/ heart edema/ heart beat defect	
	WB-4101 hydrochloride	toxic	
	(±)-Propranolol hydrochloride	mild heart edema	
	SKF 86466		
	Albuterol hemisulfate	toxic	
	SR 59230A oxalate	toxic	
	Yohimbine hydrochloride		
Angiogensis	SANT-1		
Antibiotic	**Cephalexin hydrate**		
Anticonvulsant	N-(4-Amino-2-chlorophenyl)phthalimide		
	Zonisamide sodium		
Apoptosis	Emetine dihydrochloride hydrate		
	Benzamide		
Biochemistry	1-Deoxynojirimycin hydrochloride		
	L-alpha-Methyl DOPA		
	Phosphoramidon disodium		
	N-p-Tosyl-L-phenylalanine chloromethyl ketone	toxic/ heart edema/ embryo size defect	
Ca^{2+} Channel	Phloretin		
Cell Cycle	**Ellipticine**	toxic/ heart beat defect	
	NU2058		
	Ribavirin		
Cell signaling	PD-180970	heart edema	
Cell Stress	Allopurinol		
	L-Canavanine		
	BBMP		
Cholinergic	Arecaidine propargyl ester hydrobromide	toxic	
	Bethanechol chloride		
	Edrophonium chloride		
	Choline bromide		
	**Pirenzepine dihydrochloride**		
	(-)-Scopolamine,n-Butyl-, bromide		
	Telenzepine dihydrochloride		
Cl^- Channel	**N-Phenylanthranilic acid**		
DNA	Carmustine		
	Phosphonoacetic acid		
	Hydroxyurea		
Dopamine	R(-)-2,10,11-Trihydroxy-N-propylnoraporphine hydrobromide		
	(+)-Butaclamol hydrochloride		
	Chlorprothixene hydrochloride	toxic	
	Droperidol		
	L-745,870 hydrochloride	toxic	
	Methylergonovine maleate		
	(-)-Quinpirole hydrochloride		
	(+-)-Quinpirole dihydrochloride		
	Thioridazine hydrochloride	toxic	

< Continued on next page >

Disease Models & Mechanisms • Supplementary information

GABA	PK 11195		
	CGP-13501		
	Pregnenolone sulfate sodium		
	5alpha-Pregnan-3alpha-ol-20-one	heart beat defect	
Gene Regulation	T0070907	toxic/ heart beat defect	
Glutamate	trans-(±)-ACPD		
	(±)-Ibotenic acid		
	2,6-Difluoro-4-[2-(phenylsulfonylamino)ethylthio]phenoxyacetamide	toxic/ heart beat defect	
	ATPO		
	L-Cysteinesulfinic Acid		
	Spermine tetrahydrochloride		
G-protein	U-73343	toxic	
Histamine	Diphenhydramine hydrochloride		
	Pheniramine maleate		
Hormone	AC-93253 iodide	pink head	
	17alpha-hydroxyprogesterone	heart edema	
	Cortisone		
Immune Signaling	Linezolid	toxic	
	3'-Azido-3'-deoxythymidine	heart beat defect	
	2',3'-didehydro-3'-deoxythymidine	heart edema	
	Pirfenidone	toxic	
	AFMK		
Ion Channels	**SCH-28080**		
	NS5806		
K^+ Channel	Psora-4		
	Pinacidil		
	CyPPA	toxic	
kinase phosphotase	PHA 767491 hydrochloride	toxic	
	Rhodblock 6		
Leukotriene	BWB70C		
Lipid	ET-18-OCH3		
	7,7-Dimethyl-(5Z,8Z)-eicosadienoic acid		
	CP-346086 dihydrate	toxic/ heart beat defect	
	**Atorvastatin calcium salt trihydrate**		
Multi-Drug Resistance	Sulfaphenazole		
Na^+ Channel	N-Bromoacetamide		
	Prilocaine hydrochloride		
	Tetracaine hydrochloride	toxic/edema	
Neuroscience	**RepSox**	toxic/edema/heart beat defect	
Neurotransmission	DL-alpha-Methyl-p-tyrosine		
	Hydralazine hydrochloride		
	Lithium Chloride		
Nitric Oxide	JS-K	heart beat defect	
	(±)-AMT hydrochloride		
	Aminoguanidine hydrochloride		
	3-Bromo-7-nitroindazole	toxic	
	7-Nitroindazole		
Opioid	Carbetapentane citrate		
P2 Receptor	2-Chloroadenosine triphosphate tetrasodium		

< Continued on next page >

Phosphorylation	Roscovitine		
	**PD 169316**		
	SB 415286	heart beat defect	
	ML-7	heart beat defect	
	**1-(5-Isoquinolinylsulfonyl)-3-methylpiperazine dihydrochloride**	heart edema	
	1-(5-Isoquinolinylsulfonyl)-2-methylpiperazine dihydrochloride	toxic/edema/ heart beat defect	
	LY-294,002 hydrochloride		
	Me-3,4-dephostatin	heart beat defect	
	SU 4312	yellow yolk	
	NU6027	toxic/ heart edema	
	Tyrphostin 51	heart edema	
Prostaglandin	(±)-Ibuprofen		
	(-)-Naproxen sodium		
	Phenylbutazone		
	Resveratrol		
Serotonin	CP-93129 dihydrochloride hydrate		
	Fluoxetine hydrochloride		
	Tryptamine hydrochloride		
	N-omega-Methyl-5-hydroxytryptamine oxalate salt		
	5-Hydroxyindolacetic acid		
	5-Hydroxy-L-tryptophan		
	Granisetron hydrochloride		
	L-Tryptophan		
Transcription	GW9662	heart edema	
	6(5H)-Phenanthridinone		
Tyrosine Kinase	Sunitinib malate		

Disease Models & Mechanisms • Supplementary information

Table S3. The table shows the small molecule candidates of 43 that were identified to reduce the brain hemorrhagic phenotype. The intensity of blue color represents the degree of decrease: the pale blue color indicates mild decrease while the dark blue color does a strong decrease.

**Class**	**Name**	**Symptom**	**Degree of Decrease**
Antioxidants	Sorbinil		
Adrenoceptor	p-Aminoclonidine hydrochloride		
	Bromoacetyl alprenolol menthane	heart edema	
	Imiloxan hydrochloride		
	Protriptyline hydrochloride		
Antibiotic	**Cefazolin sodium**		
	Lomefloxacin hydrochloride		
Apoptosis	Aurintricarboxylic acid		
	**4-Amino-1,8-naphthalimide**		
Bioactive Small Molecules	G15	heart edema	
Biochemistry	Acetazolamide		
	**EGTA**		
Cell Cycle	Apigenin		
Cell Stress	Lonidamine		
Cholinergic	Atropine methyl nitrate		
	Acetylthiocholine chloride		
	Oxotremorine methiodide		
Cyclic Nucleotides	Ro 20-1724		
	NS 2028		
DNA	Mitoxantrone	toxic/ heart beat defect	
Dopamine	Apomorphine hydrochloride hemihydrate		
	R(+)-6-Bromo-APB hydrobromide		
	Pergolide methanesulfonate		
Glutamate	(±)-2-Amino-3-phosphonopropionic acid		
	Chelidamic acid	toxic	
	5,7-Dichlorokynurenic acid		
	AMN082	toxic/ heart beat defect	
Histamine	(+)-Brompheniramine maleate		
	N-Methylhistaprodifen dioxalate salt		
	1-Methylhistamine dihydrochloride	toxic	
	Ranitidine hydrochloride		
Immune signaling	Maraviroc		
	3-deazaadenosine		
	Artemether	toxic	
Intracellular Calcium	Dantrolene sodium	heart edema	
Melatonin	K 185	toxic	
Na^+ Channel	Lidocaine hydrochloride		
Neurotransmission	6-Methoxy-1,2,3,4-tetrahydro-9H-pyrido[3,4b] indole		
	Chlormezanone		
	Trandolapril		
Opioid	Naloxone hydrochloride		
Serotonin	Clomipramine hydrochloride		
	1-Phenylbiguanide		

Disease Models & Mechanisms • Supplementary information

Table S4. Primers used for qRT-PCR in Supplementary figures

Name	Sequence
pcdh1g1-F	GACAGCTGGACCACGAGAAA
pcdh1g1-R	TCTGTGCCTGGTGTTGTGTT
zgc:110283-F	GCAGGAAAACCTCTGGCAGA
zgc:110283-R	ATTCTTCAGCGCCGTGATCT
pcdh2ab10-F	AAGCCCGCGTGAATGAAAAC
pcdh2ab10-R	TTTGCCTGTACTCGGAGCTC
pcdh1gc6-F	CAGTTGGAGCTCAGATTCGC
pcdh1gc6-R	CCCCTTCCCTGAATCCACAC
pcdh2g17-F	CAATGTTAACGTGGCGGTGG
pcdh2g17-R	CGCGTCTCCATCTGTAGCAT
chp2-F	GATCGACCCAAAGAGCCCAA
chp2-R	CCTGTATCGTTCGGTCTGCA
myl4-F	GCACTGGGGCTGAATCCTAC
myl4-R	CGCAAGCCCTCAACAAAGTC
zgc:162595-F	CCCGAAGATCTCAAGCCCAA
zgc:162595-R	TGGCTTTGGGGATCAGCATT
pcdh1g18-F	GAGTCGGGAGGAAAAGGCTG
pcdh1g18-R	ACACGCGCTTTTCCTGAAAC
masp2-F	AAGGCTTCCAAGTGACCCTG
masp2-R	TTTCCCAGAGCGGTCAGAAC
ldlrb-F	ACACACACTCTCCAGCATCG
ldlrb-R	CTGATCTCATGGCCCGTCAG
f7i-F	GGACACCAGGTTCCTCAAGG
f7i-R	AGATGGGCACGGCGTATAAG
capn8-F	GCTGCAGAGGAGGTTCACTT
capn8-R	CGCCATCCAGAACTCTCCAT
pcdh1g30-F	AGAGGAAATGGGCAGCGTTT
pcdh1g30-R	CCCTTGCCTAAATCCACGCT
capn2l-F	AACTGACGTCCAACCCAACA
capn2l-R	CCACATCCACCCATTCACCA
rcan1a-001-F	TTATAATGAGGAAAGTGAGGCCA
rcan1a-002-F	CACCTGAAAACAATGAAGTGCA
rcan1a-001-R, rcan1a-002-R	AATCCTCACCCGACGGAAAC
rcan1b-F	GGGCGGCGACTTCTGAAG
rcan1b-R	GCATCCGTGAAGTTGATCCG

Disease Models & Mechanisms • Supplementary information

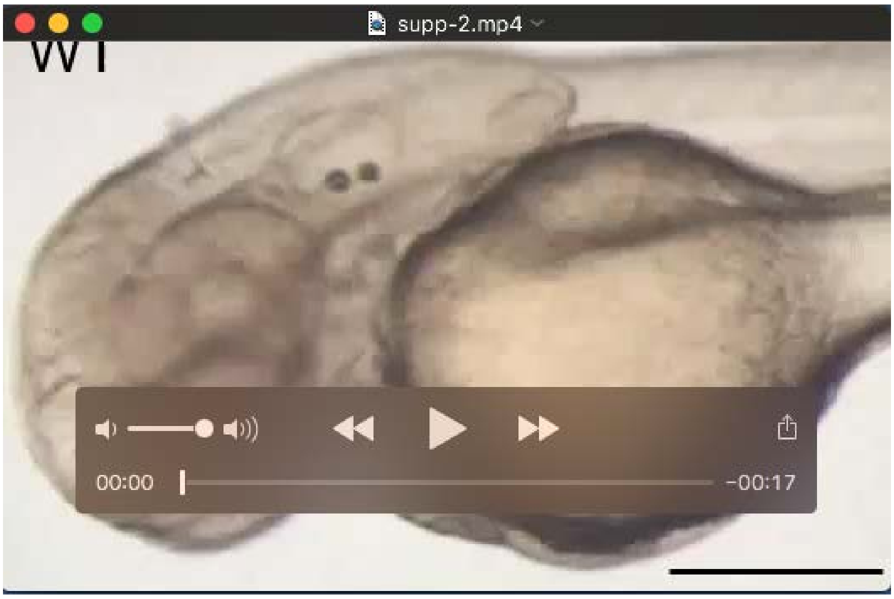

Movie 1. The video showing the heartbeat of a WT embryo at 52 hpf. Scale bar: 250 µm

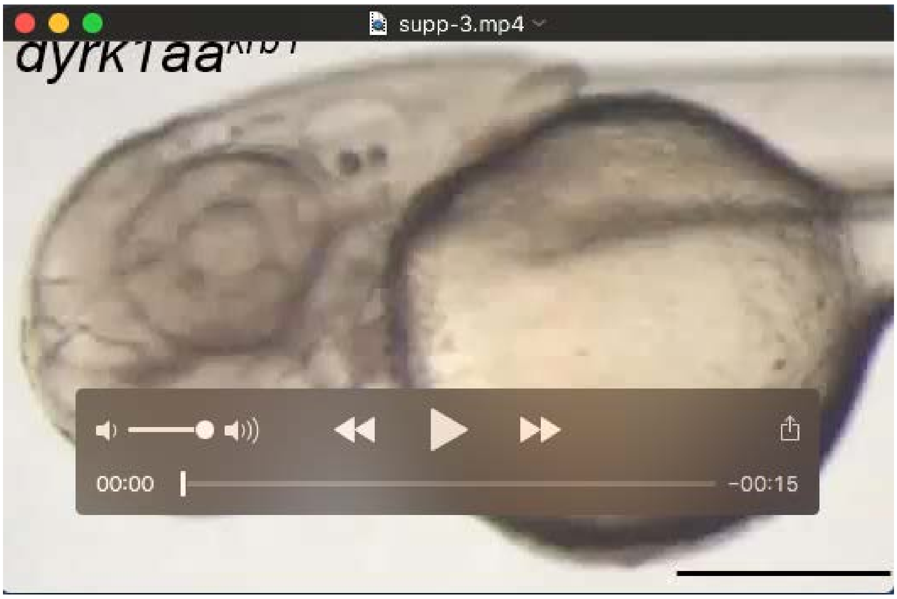

Movie 2. The video showing the heartbeat of a *dyrk1aa*^{krb1} embryo at 52 hpf. Scale bar: 250 µm

Disease Models & Mechanisms • Supplementary information
